# This thing called life

**DOI:** 10.1007/s40037-015-0201-0

**Published:** 2015-07-16

**Authors:** Anique E.N. Atherley, Charles G. Taylor

**Affiliations:** Faculty of Medical Sciences, The University of the West Indies, Cave Hill Campus, Cave Hill, Barbados

**Keywords:** Trainee well-being, Positive education, Life, Medical education

## Abstract

Academic pursuits are inseparable from the medium within which they take place — life. The lives of medical trainees can present many challenges that are independent of academic demands. Poor psychological health has been found to develop in medical trainees. Can medical educators minimize this decline in well-being? Positive education — learning skills for traditional academia and to foster happiness — has been shown to improve students’ well-being. This piece considers the application of ‘positive education’ to medical training. By using this approach, we may optimize the lives of our trainees, potentially enhance learning and improve their academic and personal outcomes.

*…We have married med[ical] students, we have students with families, we have students living on their own, paying their own bills and they have other factors in their lives that may affect them on a particular day, so you can’t tell them that “all you have to do is go home and study”.*

[Undergraduate medical student]

Medical education occurs within the context of life; learning, therefore, is inseparable. Education is one of many aspects of a medical trainee’s life and it is possible that these aspects impact each other — life may have an effect on learning. A stressful life event — divorce, grief, marriage, and depression — may strike any time during training and difficulties almost certainly will not await a lull in academic demands.

Training is intense and trainees are frequently bombarded with challenging demands with little regard for their well-being; after all, it was done to us too. Do we consider that we may be contributing to the development of poor psychological health in our trainees [1]? Some researchers have suggested that our curricula (both formal and hidden) contribute to these poor outcomes in trainees. For example, Kötter et al. described how strong consequences for absence in medical courses may lead to presenteeism — showing up to work or teaching despite illness [2] — among trainees [3]. Declines in trainees’ well-being may lower academic performance [4], increase medical errors in patient care [5], reduce life satisfaction [6] and reduce qualities of compassion, empathy and professionalism [7]. It is imperative that we do not wait until the performance of our trainees is affected before offering help. How can we help them to help themselves while learning to help others?

Students are thought to possess a ‘coping reservoir’ that is depleted by negative inputs (stress, internal conflict, demands) and replenished by positive ones (support, mentorship, intellectual stimulation) [7]. The internal structure of this reservoir is composed of individual personality traits and coping styles; this dynamic set up may lead to two outcomes: burnout or resilience [7]. We need to help our students avoid negative outcomes by maximizing their coping mechanisms and optimizing their lives and learning despite adversity. Thus far, medical educators have highlighted resilience as being key to optimizing life and learning. Resilience can be developed and learned over time [7] and encompasses the ability to bounce back from life’s challenges [8] because of an acceptance of reality, a deep belief that life is meaningful and an uncanny ability to improvise. Martin Seligman and colleagues consider resilience to be a single component of an all-encompassing concept termed ‘positive education’ [9]. Could we further optimize students’ well-being by implementing ‘positive education’ during medical training?

What exactly is ‘positive education’? This concept injects positive psychology into schooling; it is learning skills for traditional academia and to foster happiness [9]. It posits that well-being is as important to students’ development as academic learning [10]. Although much of Seligman’s research has been carried out in children and adolescents, many of the concepts are applicable to our trainees. Teaching well-being — positive education — minimizes depression, increases life satisfaction and stimulates better learning [9]. These are all outcomes that we should be desperately trying to achieve in our medical schools. If we are able to help our trainees to develop optimism, positive relationships, resilience and individual character strengths, we may optimize their journey to be lifelong learners in the intense arena of medicine. Having these skills may enable them to optimize their lives, of which education is a part. To apply positive education to medical training, we must consider both *teaching* these skills, and also *embedding* them within the curriculum [9].

It may be initially challenging for us to *teach positive education* as it is relatively new to medical training. Therefore, to demonstrate we share two techniques used by others to cultivate qualities of positive thinking and character strengths. Seligman used ‘what-went-well’ (www) exercises where students were asked to write three good things that happened to them within a specific time frame [9]. To develop character strengths, a number of tasks were carried out in Seligman’s research [9]. Firstly, students were asked to write narratives about a time they felt at their best, they were then asked to take a values in action (VIA) signature strength test [11] and lastly reflect on their narratives having discovered their signature strengths [9]. The VIA signature strengths test was developed, through positive psychology research, by Seligman and colleagues after designing a classification of 24 character strengths. Character strengths are ‘positive traits reflected in thoughts, feelings and behaviours…’ [12] and the VIA signature strengths test is a 240-item survey assessing these 24 strengths (10 items each); the test is freely available online [11]. Developing students’ assets — their character strengths — can develop resilience as they improve the resources upon which they can rely during challenges in life and learning [13]. This approach to education may allow our trainees to gain positive attributes with which to optimize life and learning [9].

While teaching positive education may be useful, Seligman speaks of reinforcement by *embedding* it into the academic curriculum [9]. This involves not simply having activities and workshops that develop students’ positive psychology, but also incorporation of positive psychology into academic learning activities [9]. Again, we understand this may be difficult to implement but it is exemplified by Seligman’s work. He describes English teachers using signature strengths and resilience to discuss English literature when reading ‘Macbeth’ [9]. Students were asked to postulate the strengths of the characters and how it aided their resilience through adversity [9]. To make this relevant to medical education, we could discuss how patients with a positive outlook achieve this resilience in the face of illness — how this is achieved through the acceptance of their reality, the belief that life is meaningful and adaptations to make the best of their situation. By teaching and embedding positivity into our curricula, our students may begin to *live positive education* as happened during Seligman’s research [9]. Participants found themselves taking positivity to their homes and everyday interactions, thus optimizing life even beyond the classroom.

No longer should we separate life and learning of our trainees; by optimizing life, we potentially optimize learning. Can our curricula contribute to our trainees’ flourishing? Maybe it is time, as Watling suggests, to consider how our curricula shape trainee well-being and rebuild them to optimize well-being [14]. We should analyze our curricula and in the same way that professional development strands exist to improve professionalism, we can add a life optimization strand. This strand could focus on nurturing positive attributes in our students such that they are more likely to develop resilience as opposed to burnout. Medical educators should consider further exploration of positive education as it may simultaneously prepare trainees for life’s challenges while enhancing academic performance.

## Funding

None.

## Conflict of interest

The authors report no declarations of interest.
